# P-487. The Clinical and Microbiological Characteristics of Surgical Site and Bloodstream Infections in Children with Heart Disease Undergoing Cardiac Surgery: A Retrospective Single-Center Study

**DOI:** 10.1093/ofid/ofaf695.702

**Published:** 2026-01-11

**Authors:** Hyeun Su Seo, Nu Ri Tchah, Hae Jung Choi, Yu Rim Shin, Jee Yeon Baek, Jong Gyun Ahn, Ji-Man Kang, Se Yong Jung, Ji Young Lee

**Affiliations:** Department of Pediatrics, Severance Children's Hospital, Yonsei University College of Medicine, Seoul, Republic of Korea, Seodaemun-gu, Seoul-t'ukpyolsi, Republic of Korea; Division of Pediatric Cardiology, Department of Pediatrics, Severance Cardiovascular Hospital, Yonsei University College of Medicine, Seoul, Republic of Korea, Seodaemun-gu, Seoul-t'ukpyolsi, Republic of Korea; Department of Pediatrics, Severance Children's Hospital, Yonsei University College of Medicine, Seoul, Republic of Korea, Seodaemun-gu, Seoul-t'ukpyolsi, Republic of Korea; Department of Thoracic and Cardiovascular Surgery, Yonsei University College of Medicine, Seoul, Republic of Korea, Seodaemun-gu, Seoul-t'ukpyolsi, Republic of Korea; Yonsei University College of Medicine, Seoul, Seoul-t'ukpyolsi, Republic of Korea; Severance Children’s Hospital, Yonsei University College of Medicine, Seoul, Seoul-t'ukpyolsi, Republic of Korea; Severance Children’s Hospital, Yonsei University College of Medicine, Seoul, Seoul-t'ukpyolsi, Republic of Korea; Division of Pediatric Cardiology, Department of Pediatrics, Severance Cardiovascular Hospital, Yonsei University College of Medicine, Seoul, Republic of Korea, Seodaemun-gu, Seoul-t'ukpyolsi, Republic of Korea; Yonsei University College of Medicine, Seoul, Seoul-t'ukpyolsi, Republic of Korea

## Abstract

**Background:**

Postoperative infections in children with heart disease are associated with increased morbidity, mortality. Appropriate empirical antibiotic selection is critical for improving outcomes. This study aimed to characterize clinical and microbiological features of postoperative infections in this population.

**Figure:**
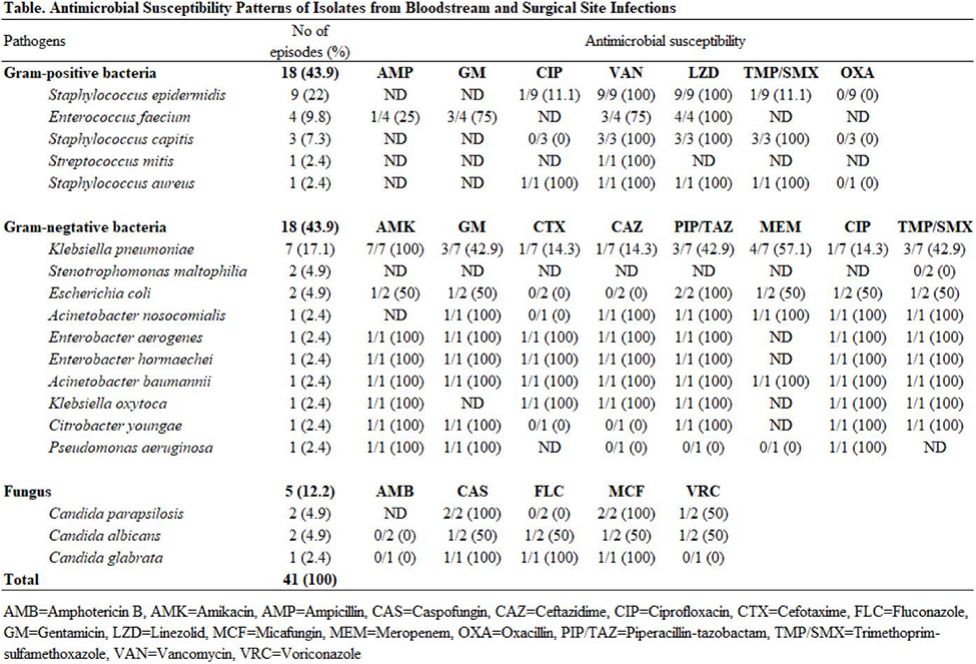

**Methods:**

This retrospective, single-center study included patients under 18years who underwent cardiac surgery or catheterization at Severance Hospital between January 1, 2020, and December 31, 2024. Isolates identified from the same type of culture more than 30 days apart were considered distinct. Primary outcome was the occurrence of microbiologically-proven bloodstream and surgical site infections (SSIs) within 30 days. Secondary outcome was to analyze the antimicrobial susceptibility.

**Results:**

A total of 1,507 procedures were performed, comprising 778 therapeutic catheterizations (51.6%), 452 simple cardiac surgeries (30.0%), and 277 complex surgeries (18.4%). 48.0% of patients were male, with a median age of 1 year and 3 months (interquartile range [IQR], 1 month–6 years and 4 months). Among cardiac surgeries (n=729), bacteremia occurred in 31 cases (4.3%), fungemia in 6 (0.8%), and SSIs in 10 (1.4%). In the catheterization group (n =778), there were 13 cases of bacteremia (1.7%), 8 of fungemia (1.0%), and 9 SSIs (1.2%). The median time to diagnosis was 7 days (IQR, 2.5–18). *Staphylococcus epidermidis* (n = 9, 20.5%) was the most common bacterial pathogen, and *Candida parapsilosis* (n = 7, 50.0%) the most common fungal isolate. All-cause mortality among patients with bloodstream or surgical site infections was 25% (12/48). Antimicrobial susceptibility results are summarized in the table.

**Conclusion:**

Bloodstream and surgical site infections following cardiac surgery in pediatric patients, though relatively infrequent, are associated with a high mortality rate of 25%. Notably, high methicillin and carbapenem resistance rates were observed. These findings emphasize the critical role of proper antibiotic use for improved patient outcome, guided by the antimicrobial stewardship program.

**Disclosures:**

All Authors: No reported disclosures

